# Supplementary figures and images for: Differential polarization of cortical pyramidal neuron dendrites through weak extracellular fields
Source: PLoS Comput Biol. 2018 May 4;14(5):e1006124. doi: 10.1371/journal.pcbi.1006124 (PMC5955601; doi:10.1371/journal.pcbi.1006124)

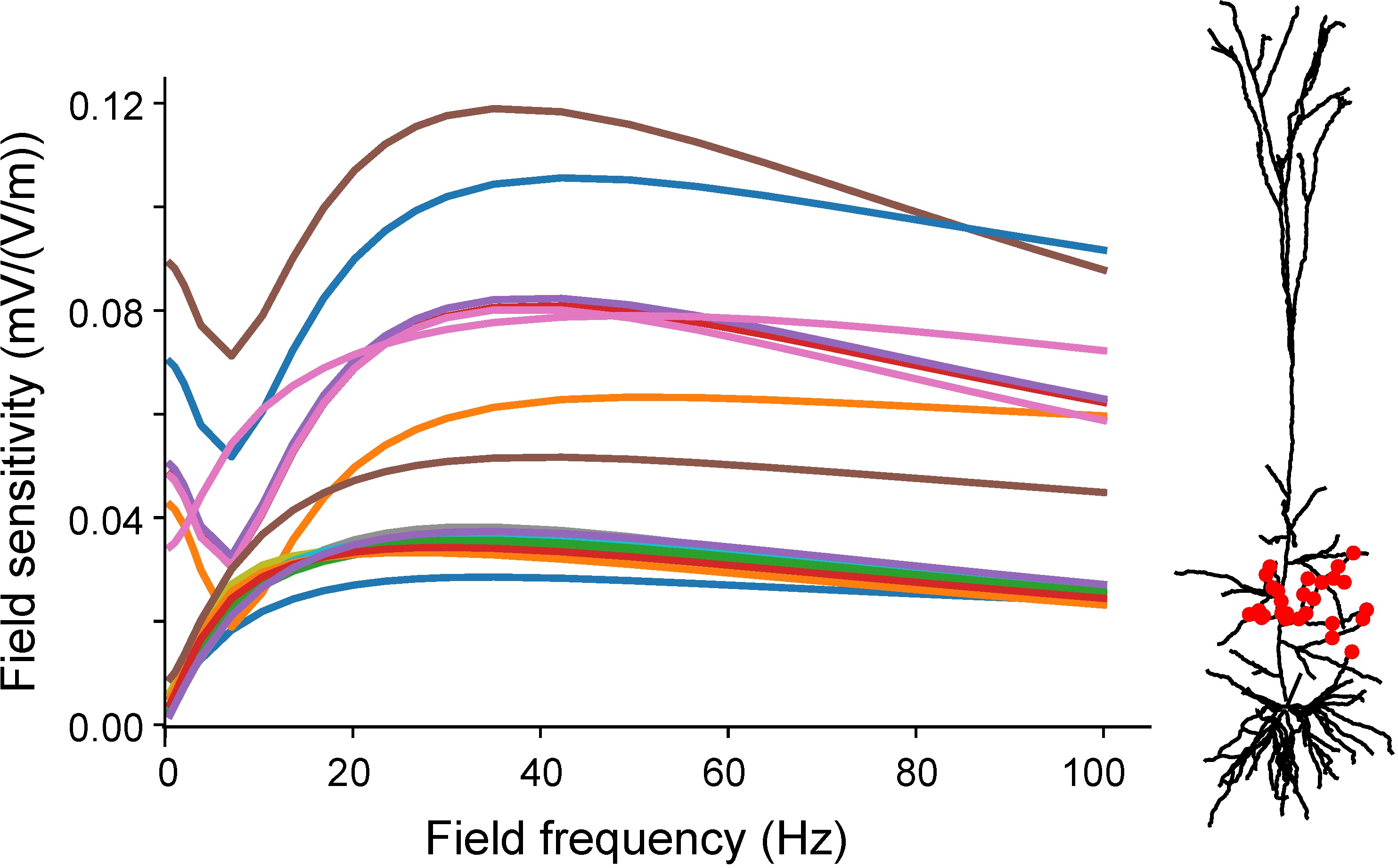

Supplement: S1 Fig — (Left) Frequency-dependent sensitivity of the passive cell, i.e. without any active ion channels, due to AC field parallel to the somato-dendritic axis. The locations where these sensitivities are measured are displayed on the cell (right). (TIF) [file pcbi.1006124.s001.tif]

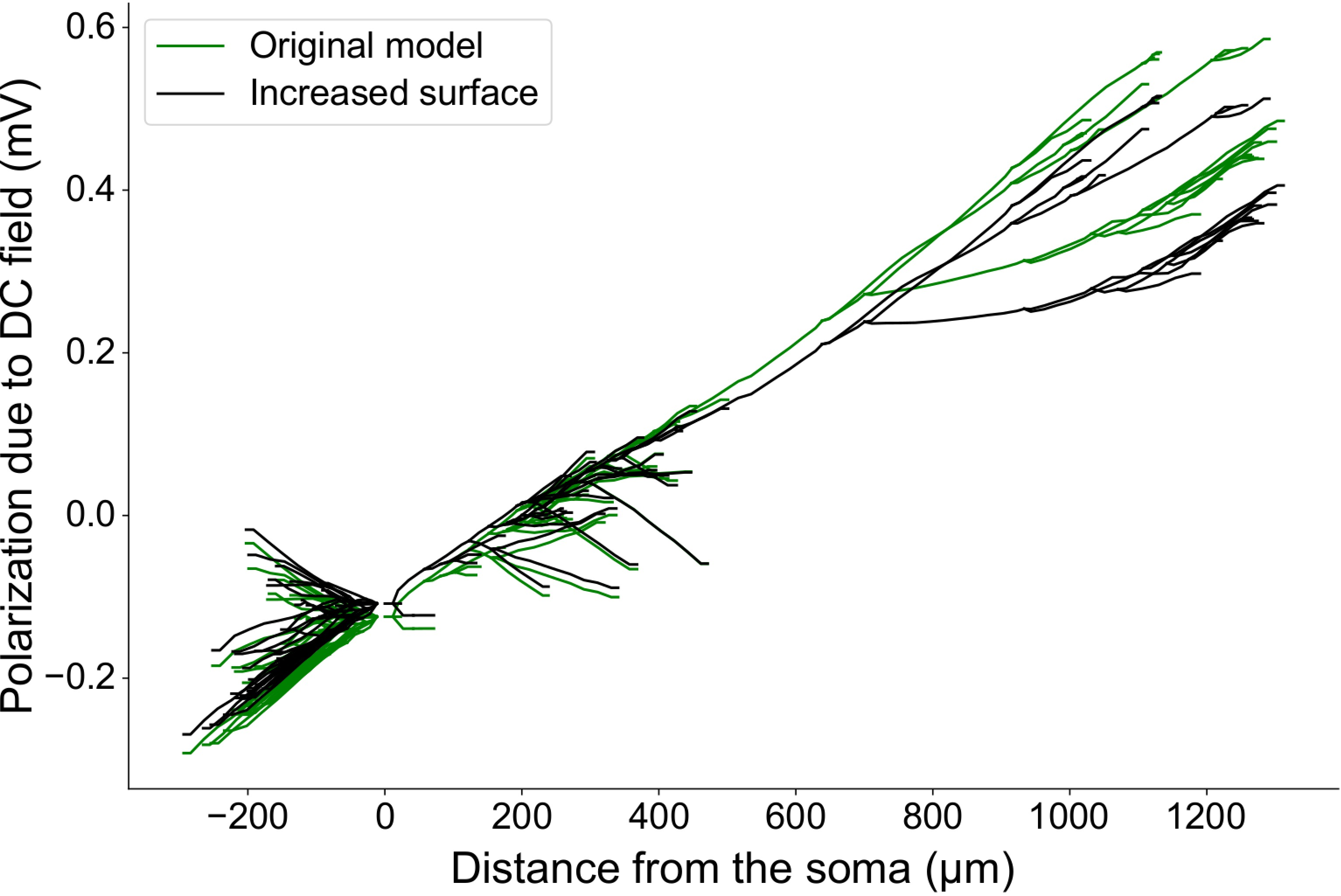

Supplement: S2 Fig — Polarization of passive cells due to a positive 1 V/m field plotted as the function of the distance from the soma. For clarity basal dendrites are plotted with negative distance. The polarization is plotted for original cell (green) and for a cell with an increased membrane surface (black). The passive cell properties of the original cell, e.g. membrane conductance and capacitance, are the one reported in Hay et al. 2011 [16]. In the other cell, we mimic a 40% increase of the membrane surface by adjusting the membrane conductance and capacitance [29, Chapter 4.3.2] (see Methods). (TIF) [file pcbi.1006124.s002.tif]

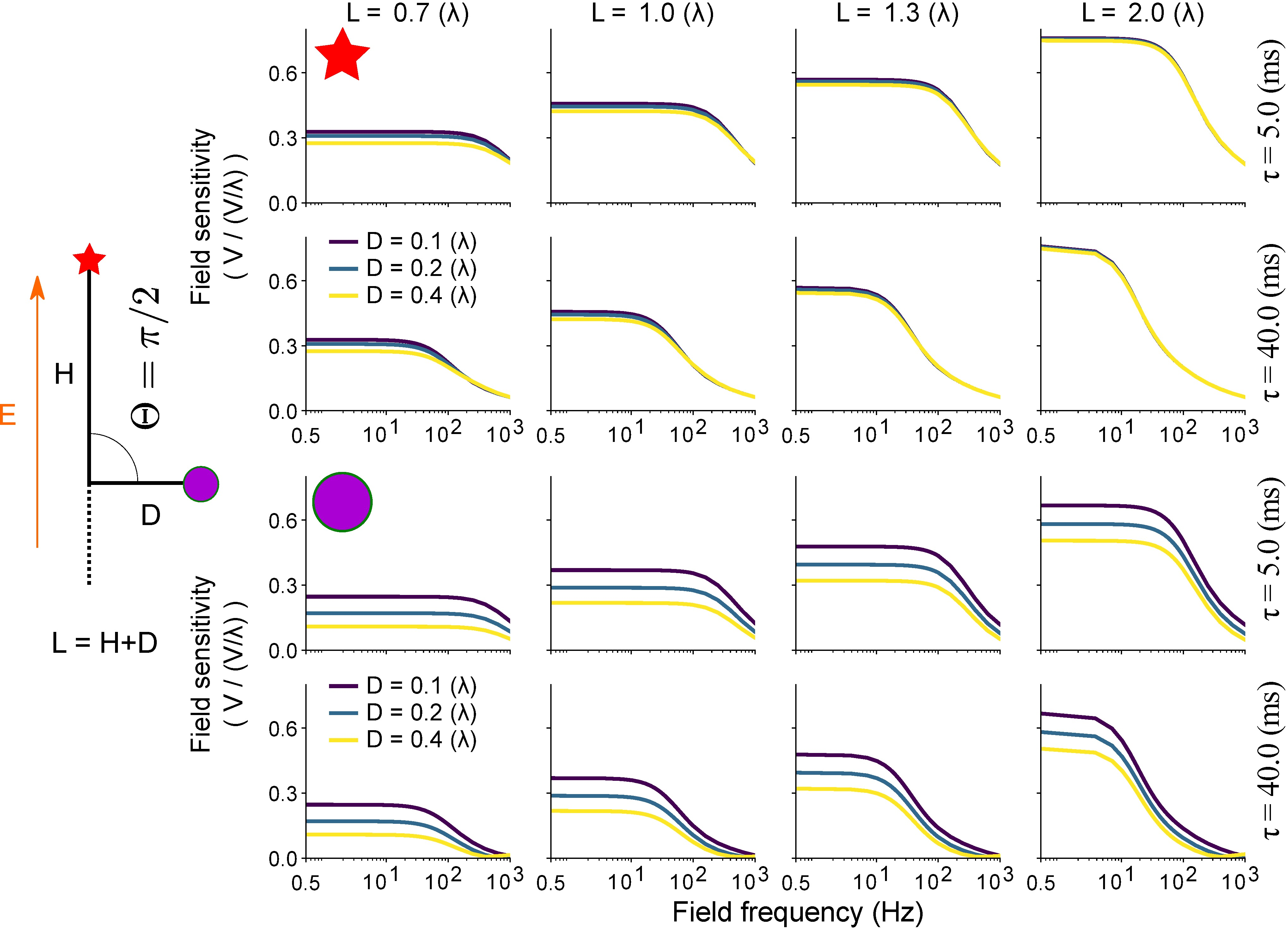

Supplement: S3 Fig — The subplots represent the field sensitivity (in V / (V/λ)) at both cable ends: (top,red star) the unbent branch and (bottom, violet circle) the bent one, as function of the field frequency (x axis). The field sensitivity are displayed for various membrane time constant τ (rows for each location), total cable length L (increasing from left to right) and bent branch length D (color coded). The bending angle is Θ = π/2 (rad). (TIF) [file pcbi.1006124.s003.tif]

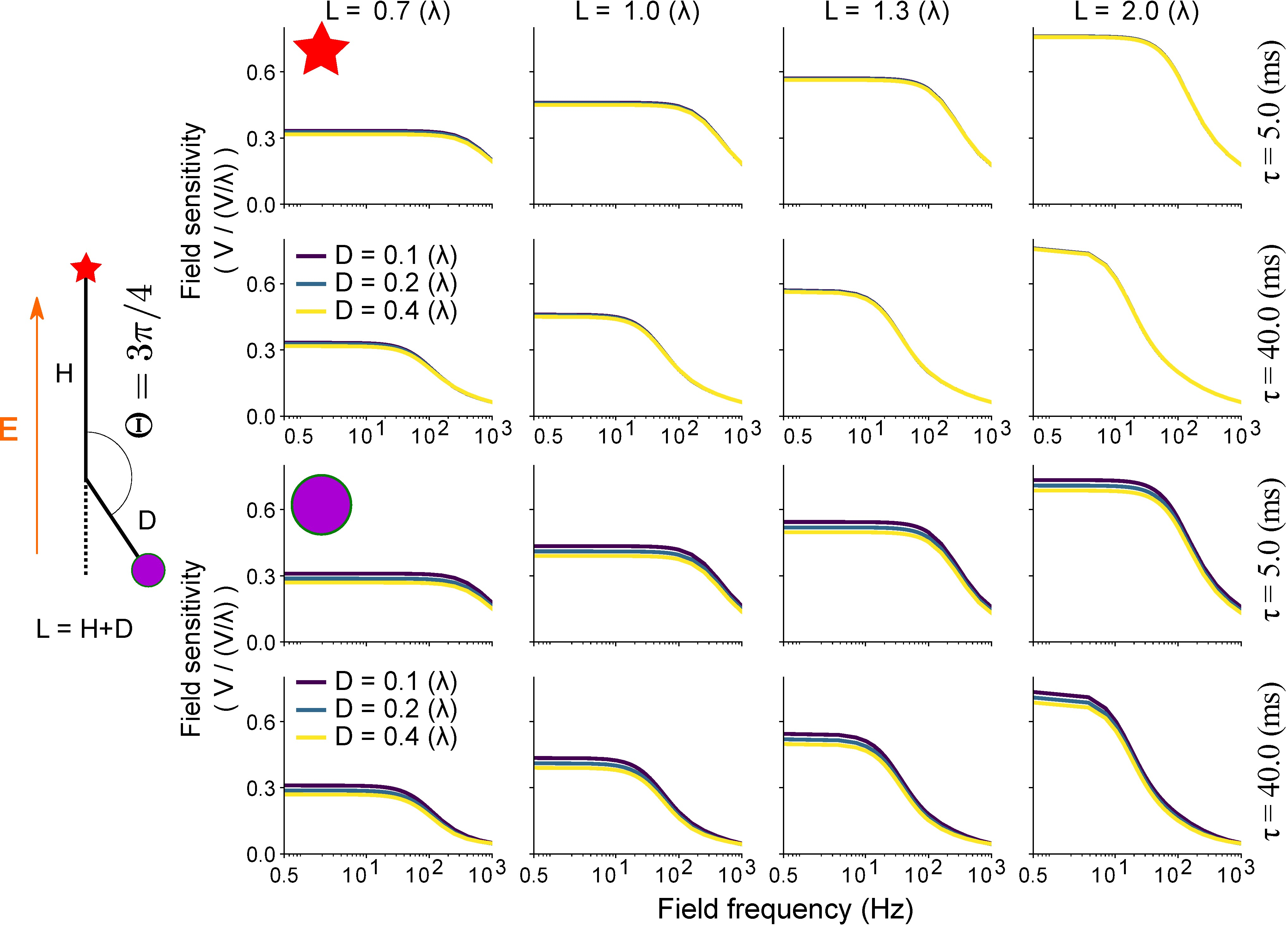

Supplement: S4 Fig — The subplots represent the field sensitivity (in V / (V/λ)) at both cable ends: (top,red star) the unbent branch and (bottom, violet circle) the bent one, as function of the field frequency (x axis). The field sensitivity are displayed for various membrane time constant τ (rows for each location), total cable length L (increasing from left to right) and bent branch length D (color coded). The bending angle is Θ = 3π/4 (rad). (TIF) [file pcbi.1006124.s004.tif]

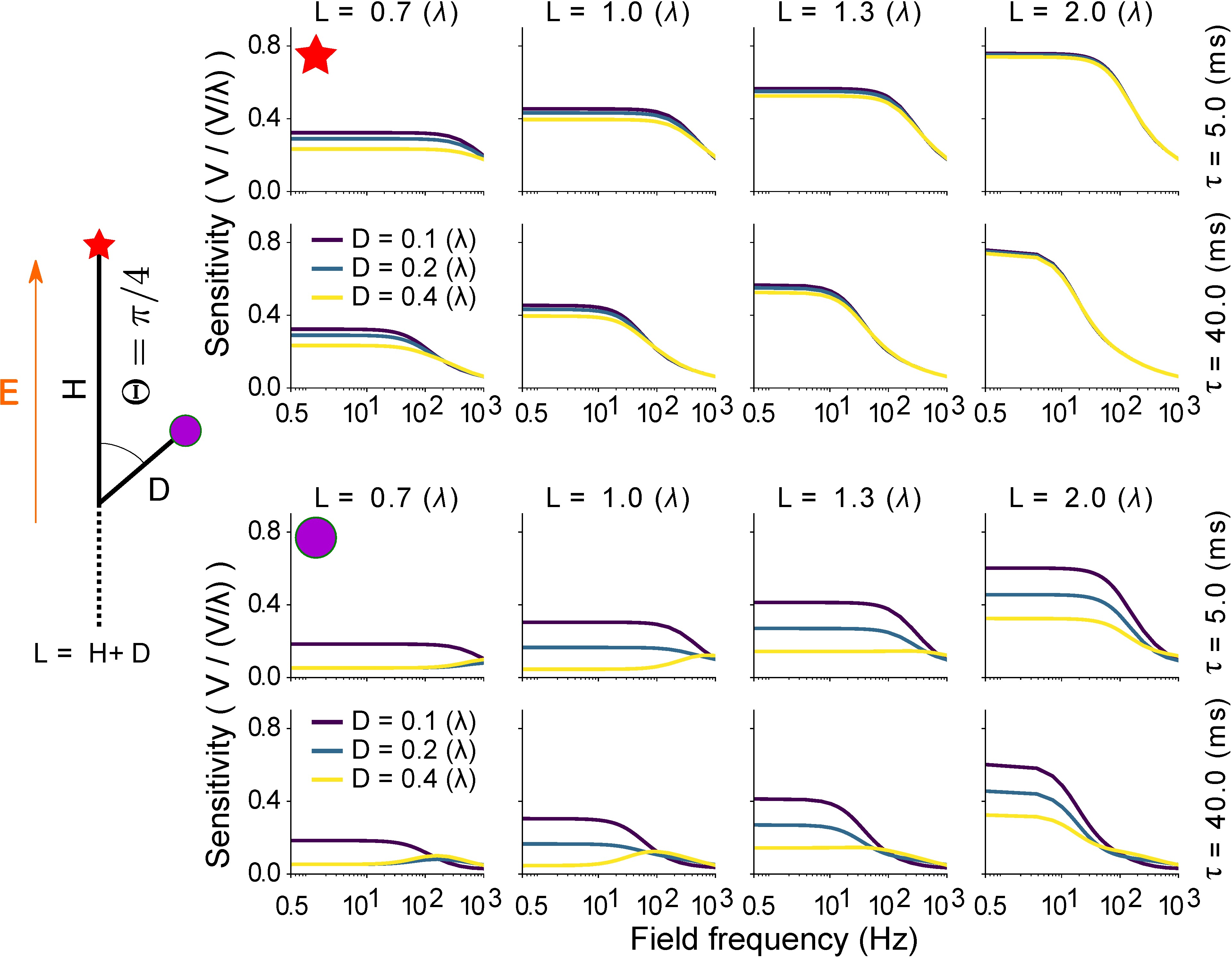

Supplement: S5 Fig — The subplots represent the field sensitivity (in V / (V/λ)) at both cable ends: (top,red star) the unbent branch and (bottom, violet circle) the bent one, as function of the field frequency (x axis). The field sensitivity are displayed for various membrane time constant τ (rows for each location), total cable length L (increasing from left to right) and bent branch length D (color coded). The bending angle is Θ = π/4 (rad). (TIF) [file pcbi.1006124.s005.tif]

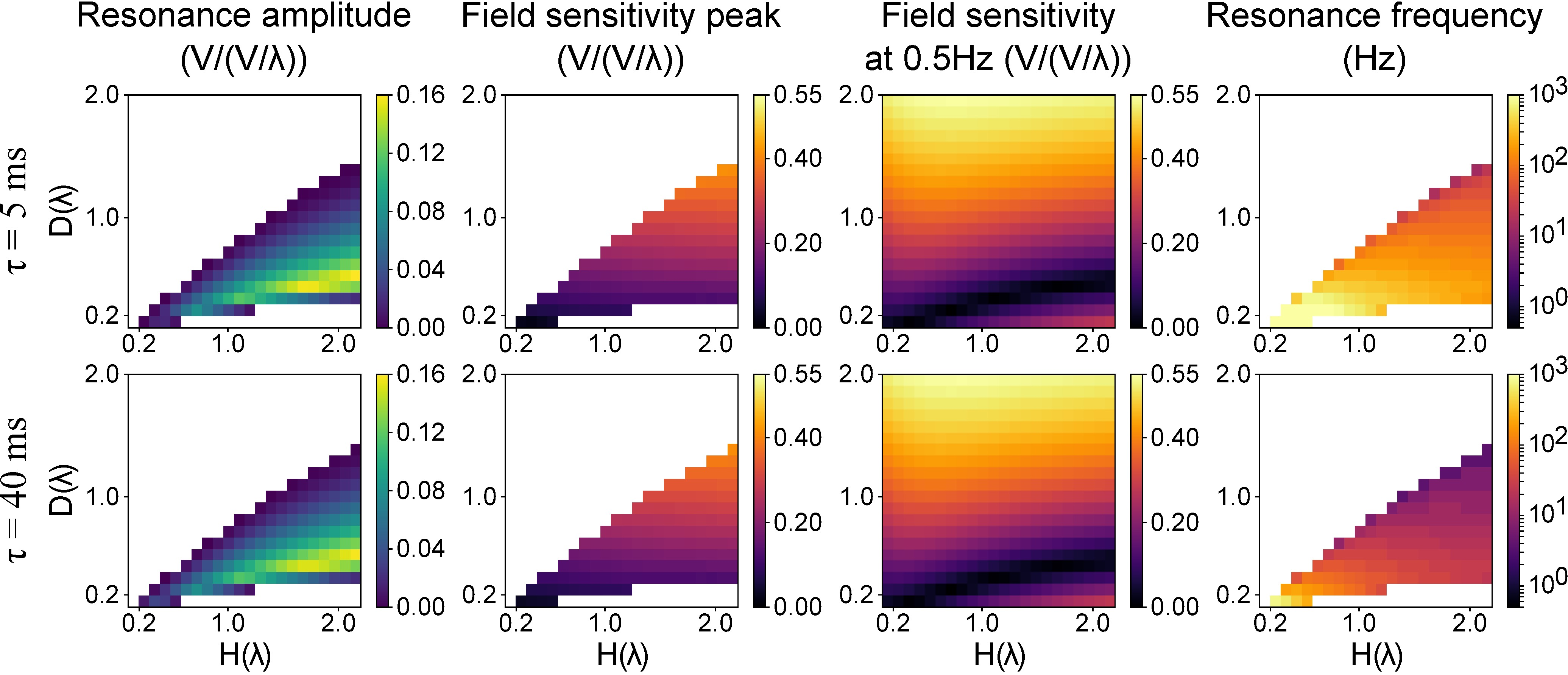

Supplement: S6 Fig — We consider the sensitivity to AC fields of a cable with acute bending angle (Θ = π/4) measured at the bent extremity. From left to right, the plots represent the resonance amplitude (peak field sensitivity, i.e. at the resonance, minus the field sensitivity at 0.5Hz), the peak field sensitivity, the field sensitivity at 0.5 Hz and the resonance frequency. Each of these measures is plotted for various electrotonic lengths of the main branches, H, (vertical axis) and the bent branch electrotonic length, D (horizontal axis). The white areas correspond to the absence of resonance. The rows correspond to different membrane time constants (top: τ = 5 ms, bottom: τ = 40 ms). (TIF) [file pcbi.1006124.s006.tif]

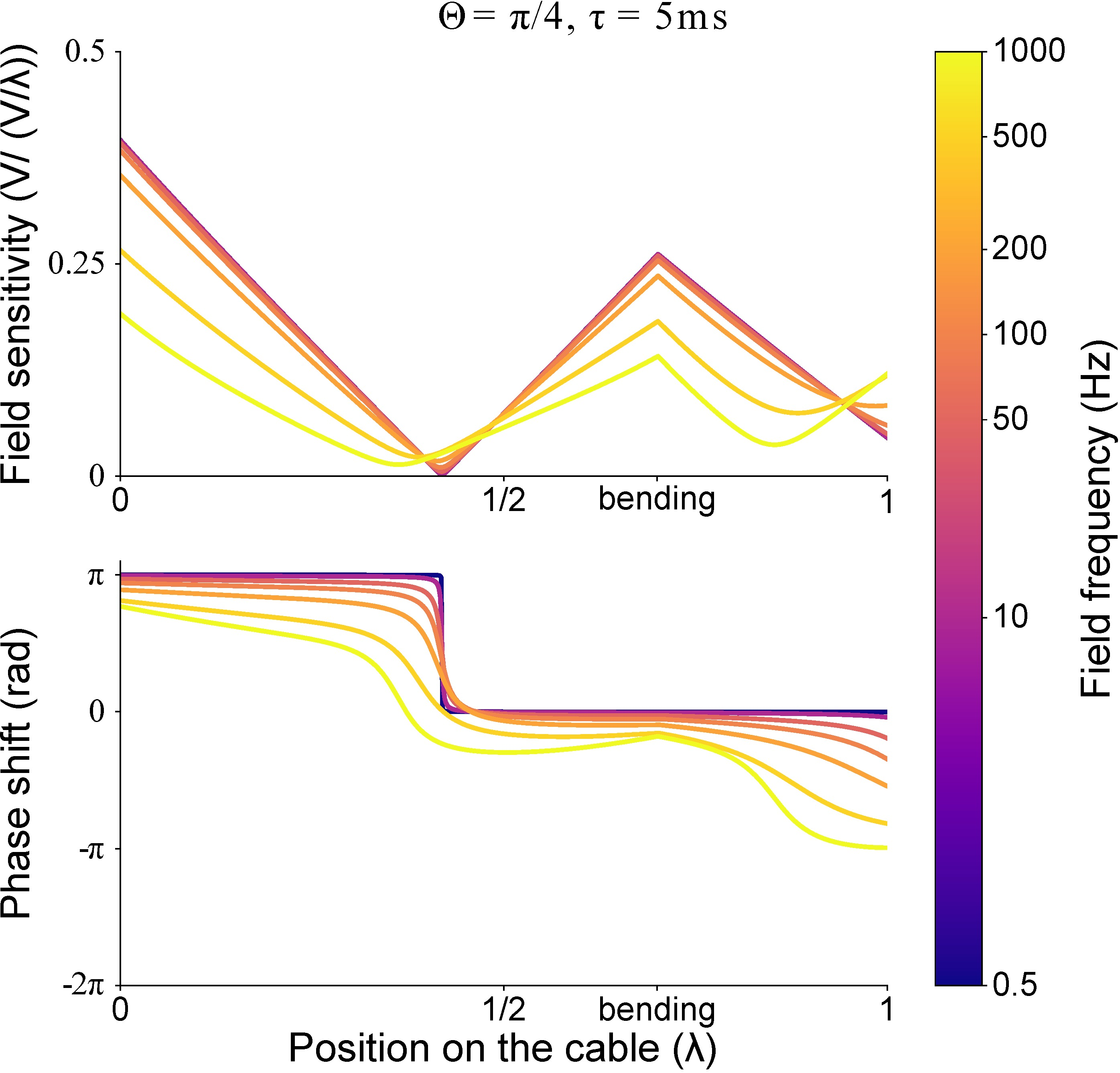

Supplement: S7 Fig — Distribution of the sensitivity (top) and phase (bottom) along the bent cable for different field frequencies (0.5, 10, 50, 100, 200, 500 and 1000Hz). Cable parameters are τ = 5(ms), L = 1(λ), Θ = π/4 (rad) and D = 0.4(λ). (TIF) [file pcbi.1006124.s007.tif]

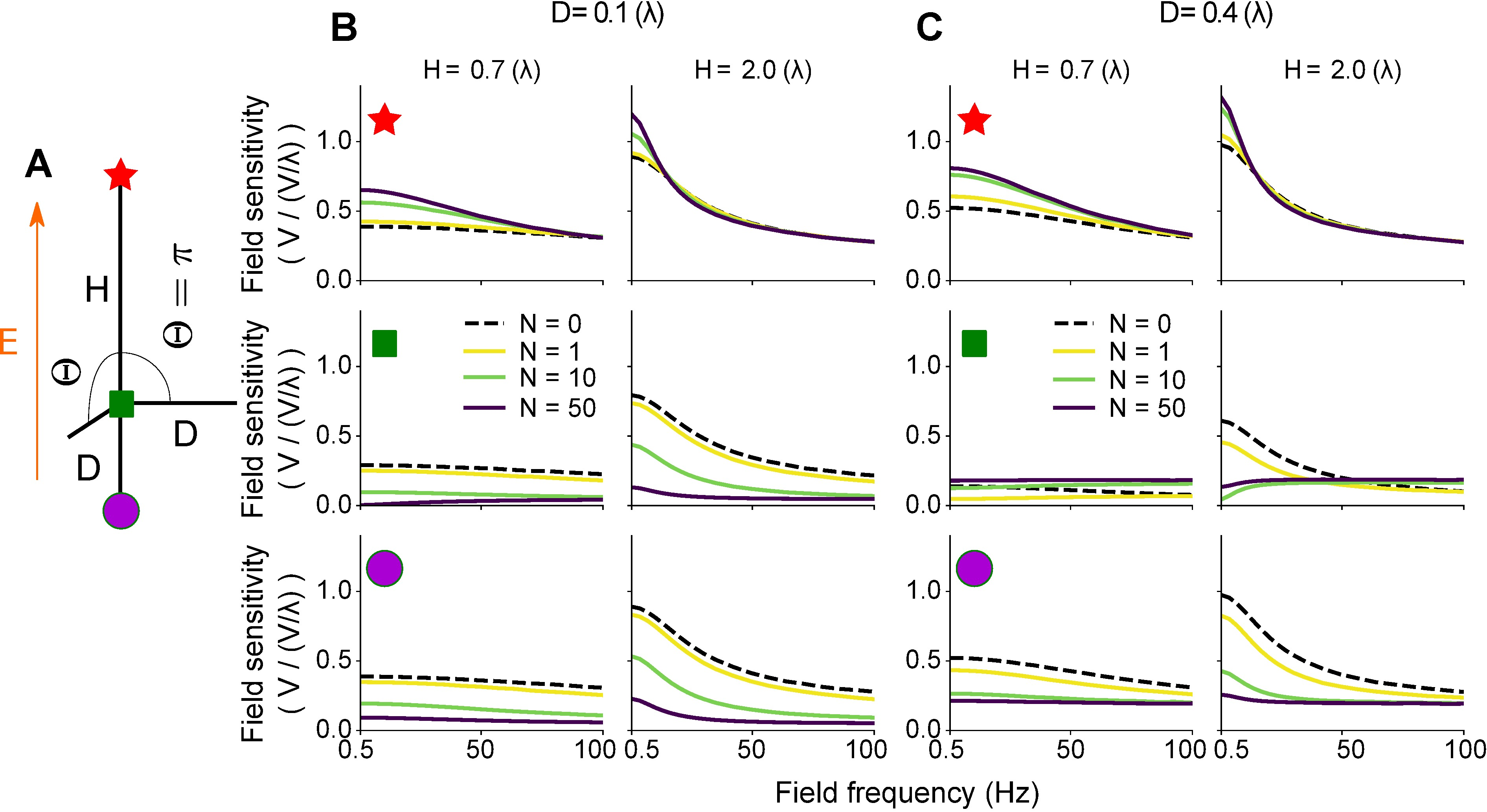

Supplement: S8 Fig — (A) Schematic representation of the simplified neuron model at use. The model consists in a main branch of length H, parallel to the extracellular field. Several branches of length D are attached to one end of the main cable. At least one of the attached branch is parallel to the main cable axis, the others form an angle Θ with that axis. (B,C) Sensitivity (in V/(V/λ)) at the end of the main cable (red star), at the branching point (green square) and at the end of the parallel branching cable (purple circle), as function of the field frequency. The field sensitivities are displayed for various number N (color coded) of branches with an angle of Θ and various main cable’s (H, columns) and branches (D, columns) lengths. In all the plots the bending angle is Θ = π (rad) and the membrane time constant τ = 40(ms). H and D are electrotonic lengths. (TIF) [file pcbi.1006124.s008.tif]

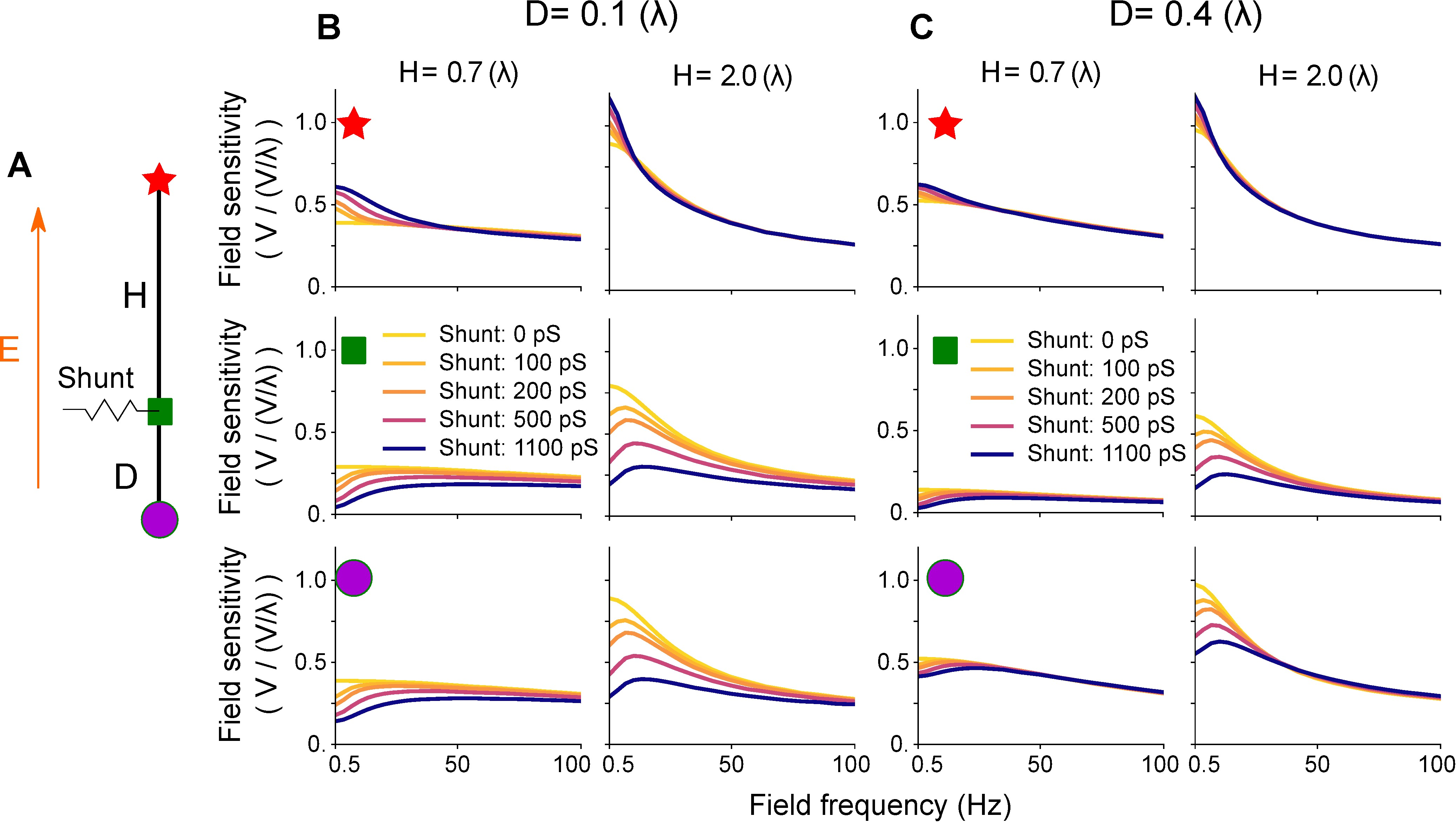

Supplement: S9 Fig — (A) Schematic representation of the simplified neuron model at use. The model consists in a passive cable with a local shunt (green square), i.e. an additional local conductance. The shunt is located at a distance H from one cable end and D from the other; H being longer than D. The cable is subject to an extracellular field parallel to it. (B,C) Sensitivity (in V/(V/λ)) at both cable ends (red star and purple circle) and at the location of the shunt (green rectangle), as function of the field frequency. The sensitivities are displayed for various shunt conductances (color coded) and various distances between the shunt and both extremities (H and D columns). In all the plots the membrane time constant is τ = 40(ms). H and D are electrotonic lengths. (TIF) [file pcbi.1006124.s009.tif]

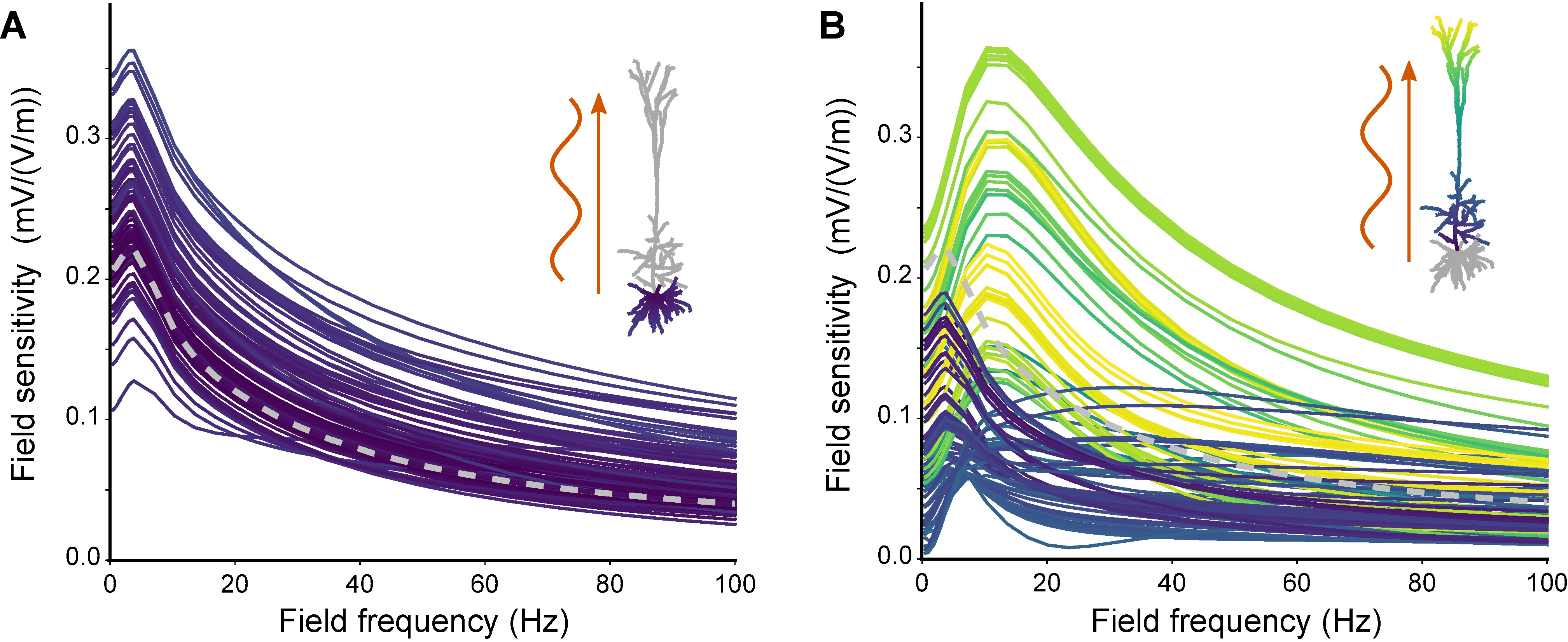

Supplement: S10 Fig — Frequency-dependent sensitivity of the cell to AC field measured at different locations at the basal (A) or apical (B) dendrites. Colors code the distance to the soma of the considered segment. (TIF) [file pcbi.1006124.s010.tif]

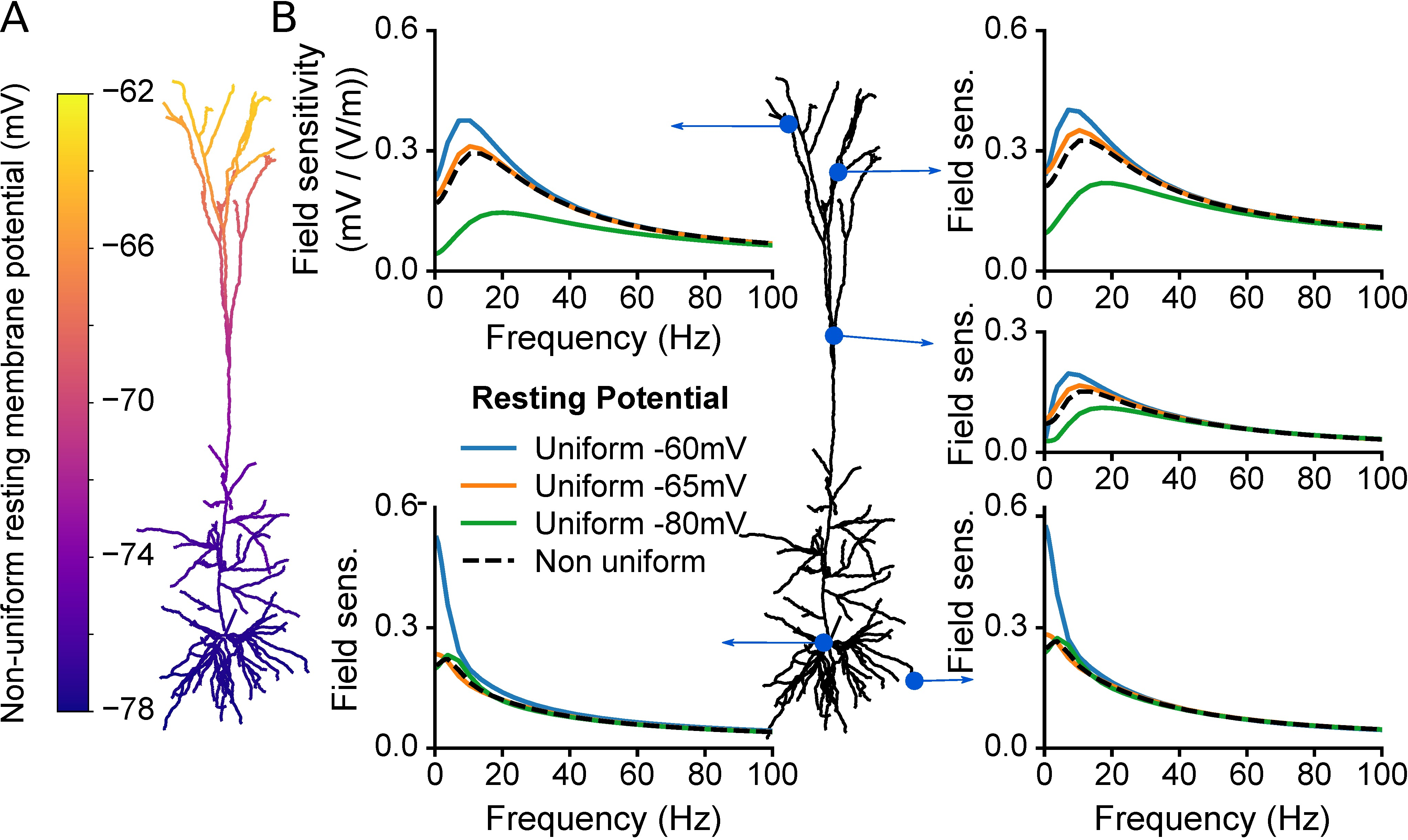

Supplement: S11 Fig — (A) Distribution of the membrane potential at rest, i.e. in absence of electrical fields, in the fully active Hay et al. model. (B) Field sensitivity of the fully active model in case of non-uniform and uniform resting membrane potential. The uniform distribution of the resting membrane was set to an arbitrary value by adjusting the passive leak reversal potential at each dendritic segment independently (see Methods). (TIF) [file pcbi.1006124.s011.tif]

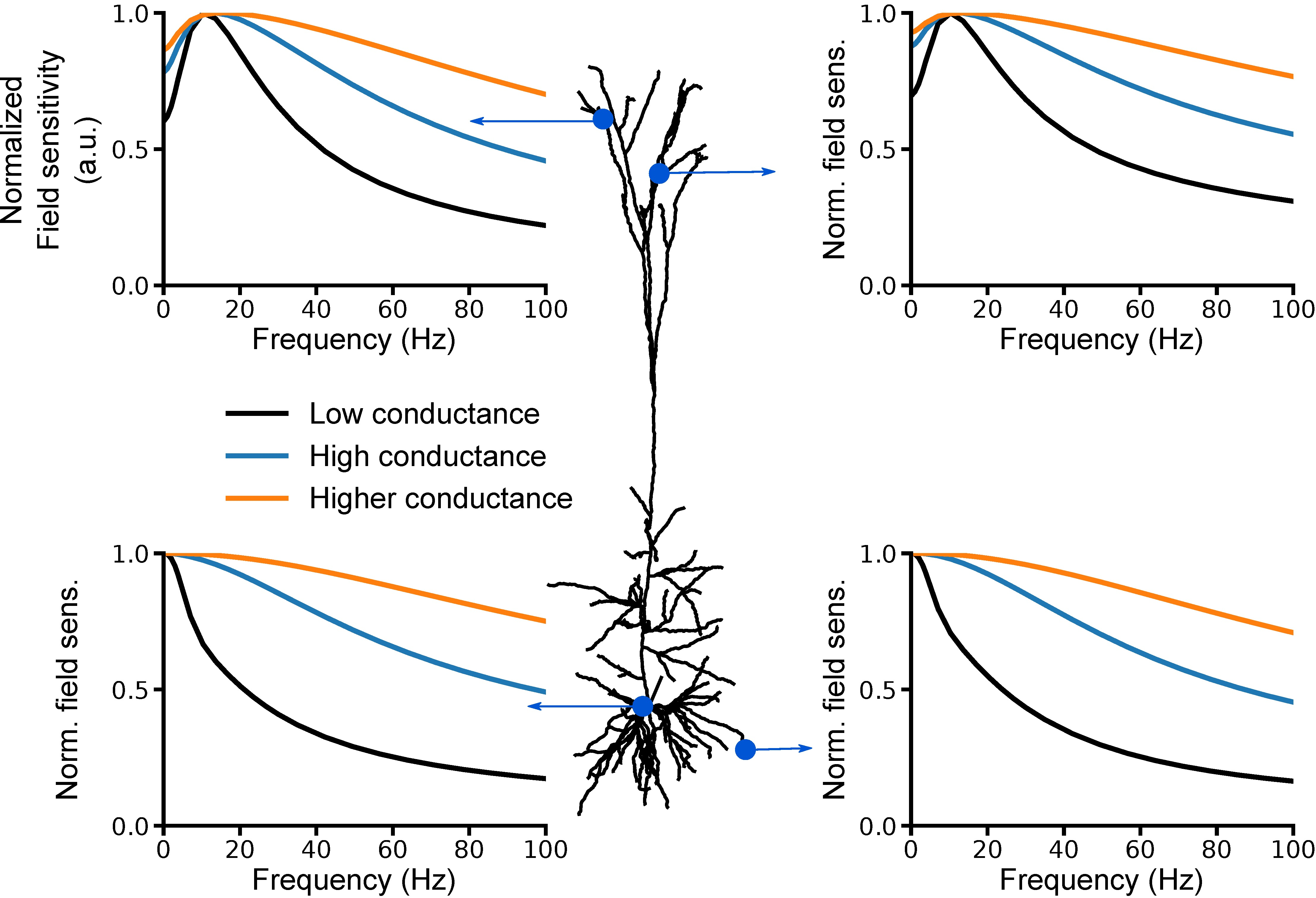

Supplement: S12 Fig — We consider the field sensitivity of a neuron model in different conductance states. The low-conductance state corresponds to the model with the same leak conductance as the original Hay et al. [16] model. In the high- (blue lines) and higher-conductance (orange lines) state, we uniformly increase the passive conductance of the original model by adding respectively 350μS/cm2 and 900μS/cm2. To remove the effects of changes in resting membrane potential, we uniformly set the resting membrane potential of all 3 models to -65mV by adjusting the leak reversal potential (see Methods). The resistance to somatic input current of the low-, high- and higher-conductance state model are respectively: 53 MΩ, 15.62 MΩ, and 9.05 MΩ The subplots display the normalized field sensitivity (in fraction of the local peak sensitivity) for the models in different conductance state. (TIF) [file pcbi.1006124.s012.tif]

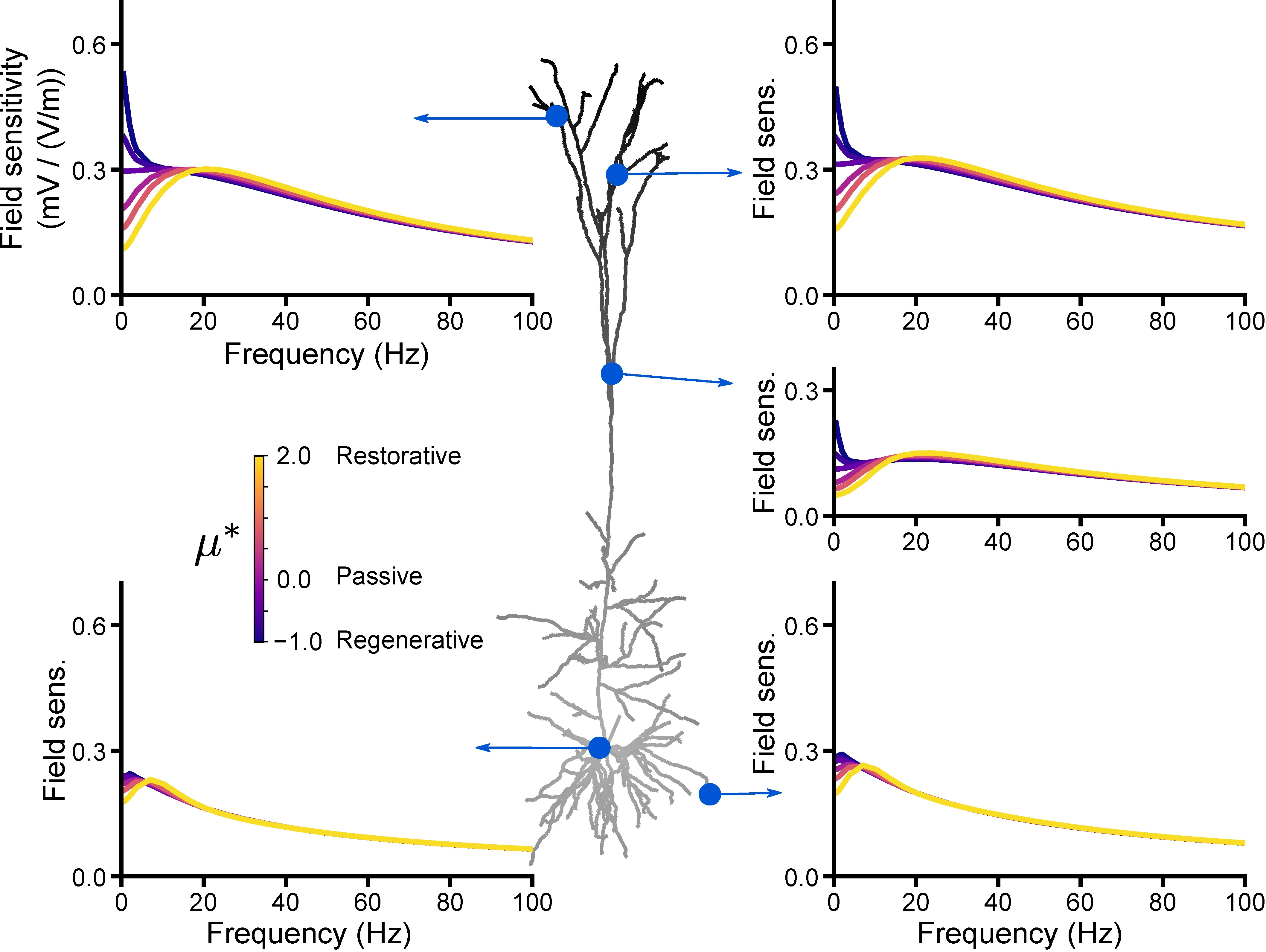

Supplement: S13 Fig — The neuron model includes a leak current and a single QA channel, whose conductance distribution increases linearly with distance from the soma. The shades of grey in the cell plot represent this distribution. μ* determines the type of the QA channel. The plots displays the cell’s sensitivity to AC fields at different locations depending on the values of μ* (color coded). (TIF) [file pcbi.1006124.s013.tif]

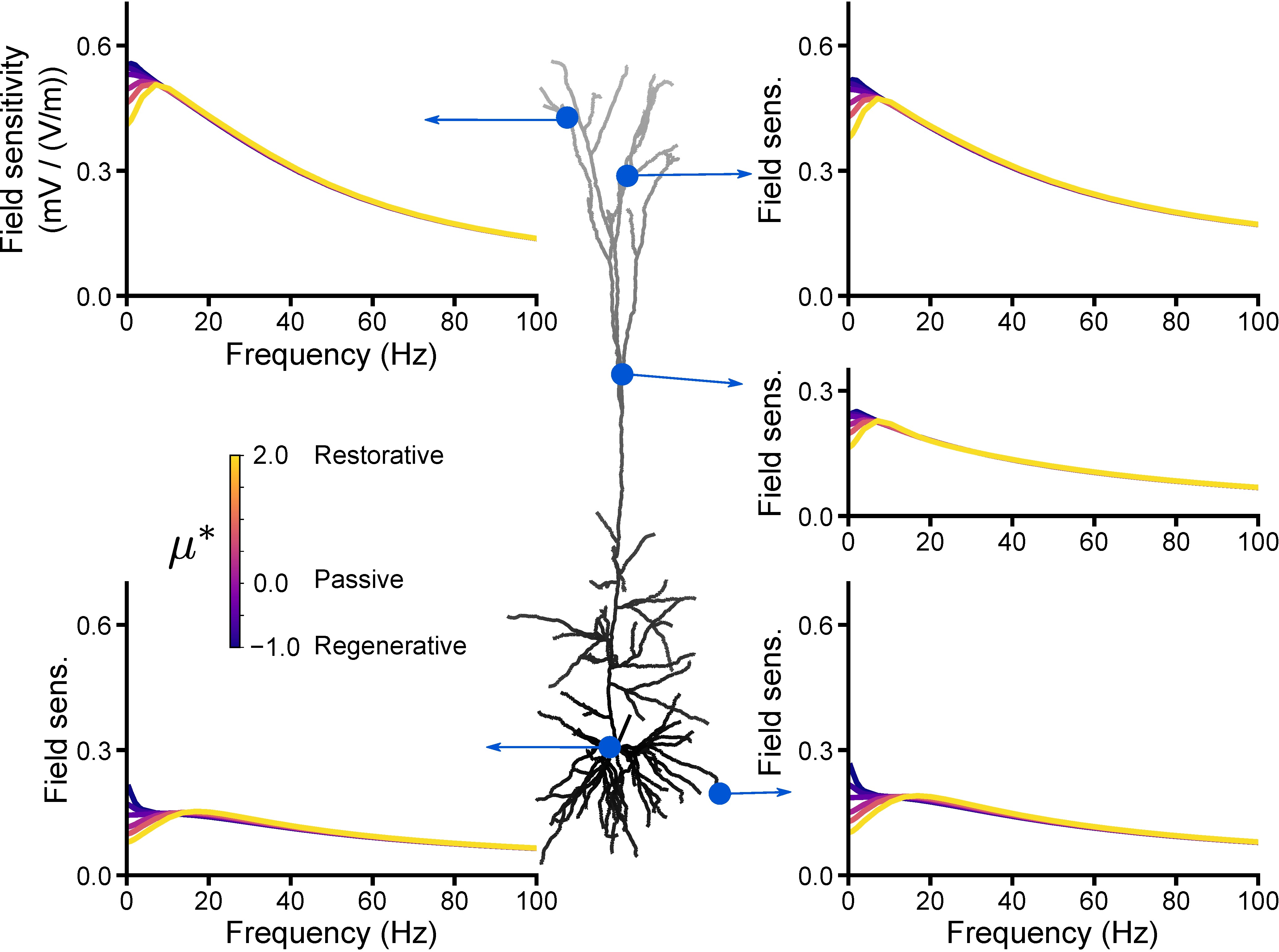

Supplement: S14 Fig — The neuron model includes a leak current and a single QA channel, whose conductance distribution decreases linearly with distance from the soma. The shades of grey in the cell plot represent this distribution. μ* determines the type of the QA channel. The plots displays the cell’s sensitivity to AC fields at different locations depending on the values of μ* (color coded). (TIF) [file pcbi.1006124.s014.tif]

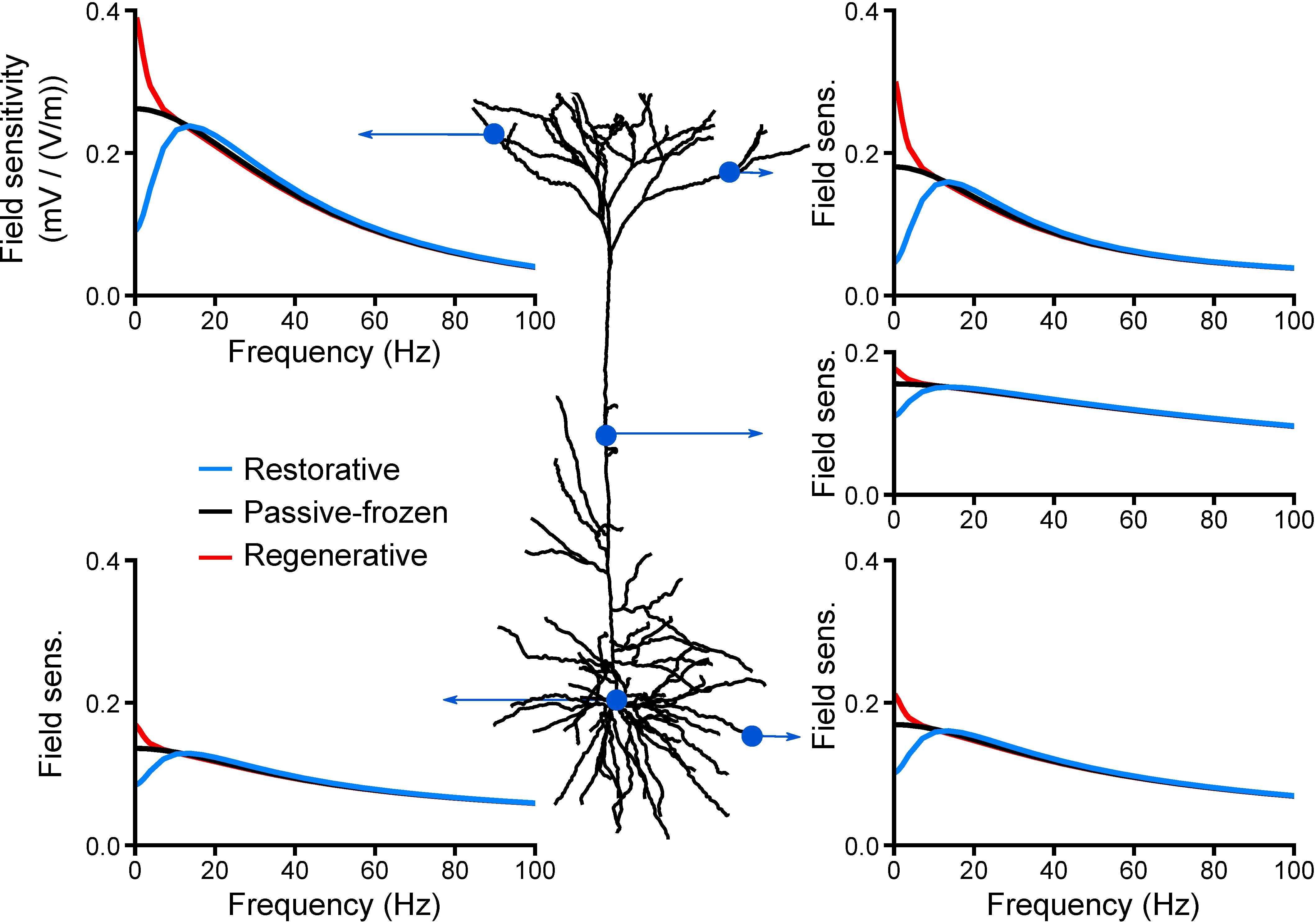

Supplement: S15 Fig — We consider a neuron model which includes solely a leak conductance and a single uniformly distributed quasi-active channel (QA). We use the reconstructed morphology corresponding to cell 2 in the Hay et al. [16] paper. The plots display the cell’s sensitivity (in mV/(V/m)) to AC fields at different locations in case of restorative (blue, μ* = 2), passive (black, μ* = 0) and regenerative (red, μ* = −0.5) quasi-active currents. (TIF) [file pcbi.1006124.s015.tif]

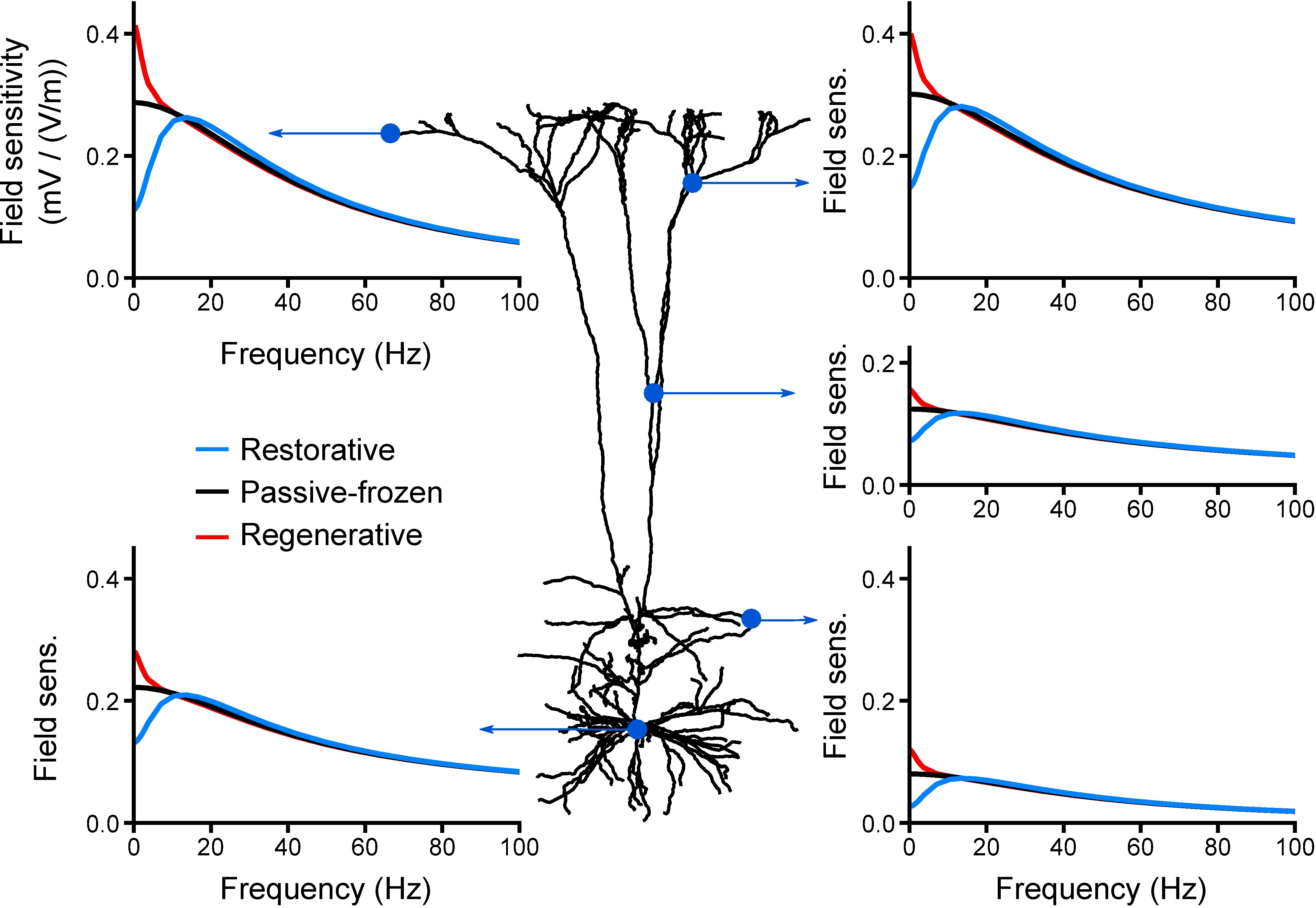

Supplement: S16 Fig — We consider a neuron model which includes solely a leak conductance and a single uniformly distributed quasi-active channel (QA). We use the reconstructed morphology corresponding to cell 3 in the Hay et al. [16] paper. The plots display the cell’s sensitivity (in mV/(V/m)) to AC fields at different locations in case of restorative (blue, μ* = 2), passive (black, μ* = 0) and regenerative (red, μ* = −0.5) quasi-active currents. (TIF) [file pcbi.1006124.s016.tif]

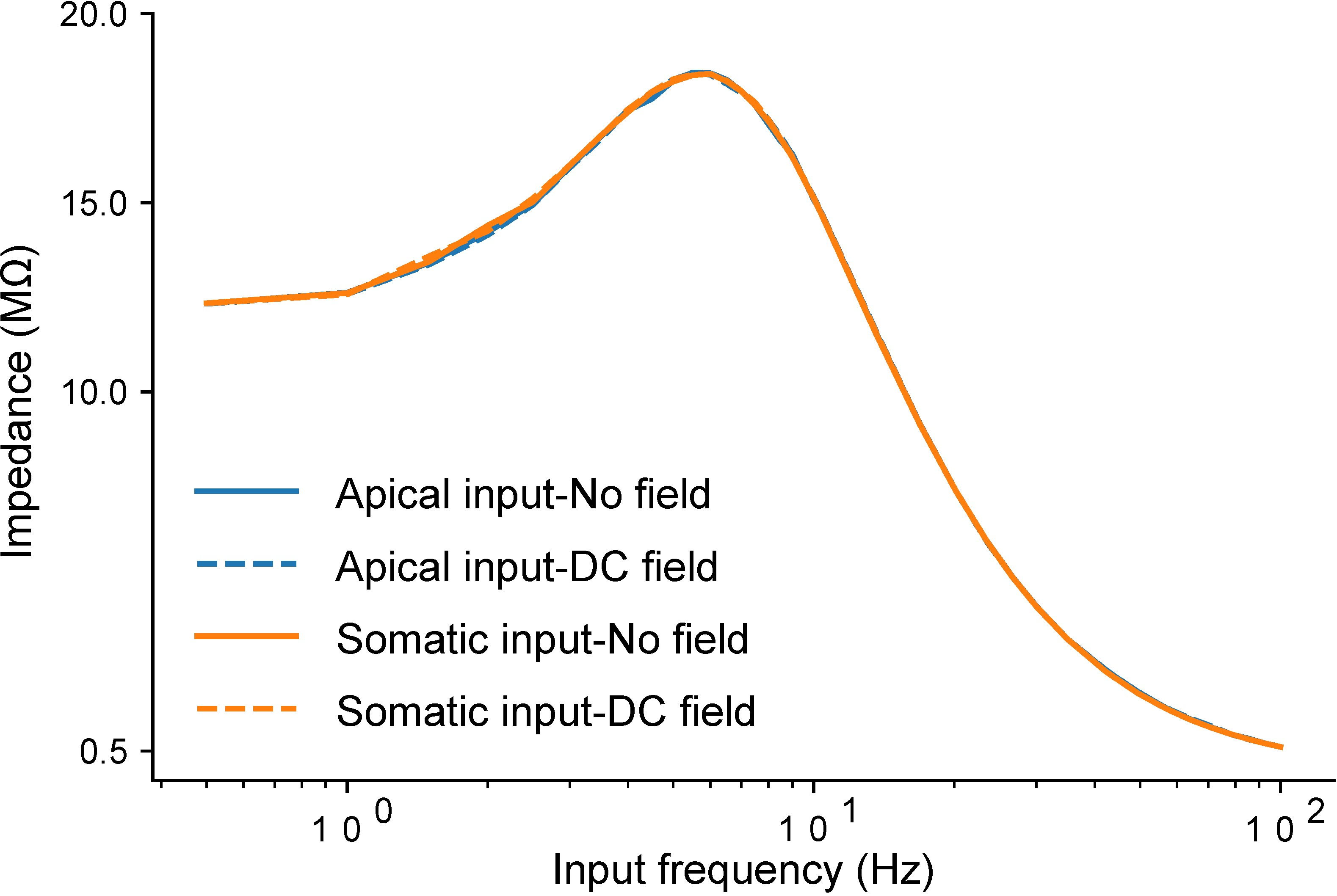

Supplement: S17 Fig — The impedances are displayed in the absence of field (solid lines) and in the presence of a positive DC field of 1 V/m (dashed lines). The impedances are computed through the injection of a low amplitude sinusoidal current at one location and measuring the membrane potential response at the other. The amplitude of the injected current is 0.05 nA for an injection at the soma and 0.01 nA for a dendritic injection. The apical location is located at 620 μm from the soma. (TIF) [file pcbi.1006124.s017.tif]
